# Advancements in technique and technology of minimally invasive and endoscopic spine surgery

**DOI:** 10.3171/2024.1.FOCVID23218

**Published:** 2024-04-01

**Authors:** Mark A. Mahan, Hyeun-Sung (Harrison) Kim, Laura A. Snyder, Richard G. Fessler

**Affiliations:** 1 Department of Neurosurgery, University of Utah, Salt Lake City, Utah;; 2 Department of Neurosurgery, Nanoori Hospital Seoul, Seoul, Republic of South Korea;; 3 Department of Neurosurgery, Barrow Neurological Institute, Phoenix, Arizona; and; 4 Department of Neurosurgery, Rush University Medical Center, Chicago, Illinois

Innovation is the lifeblood of surgery. We strive for both incremental and revolutionary improvement in patient outcomes—from ourselves in developing techniques as well as from technology that enables surgery. This issue of *Neurosurgical Focus: Video* is a testament to both fronts, describing the expansion of surgical techniques in more complex realms, as well as novel systems for improving our surgical success.

Minimally invasive spine surgery has delivered on its promise of improved patient care—offering less muscle trauma, quicker recovery, and oftentimes fewer risks. It has also enabled new venues for surgery, which have reduced societal costs. Endoscopic spine surgery further minimizes approach-related trauma and some elements of risk reduction, such as near elimination of postoperative infections.^1^

Our field is undergoing an immense unfolding of the opportunities of minimally invasive and endoscopic spine surgery. We are witnessing a burgeoning of systems and approaches for more surgical lesions. Herein, readers will be able to review approaches for pathologies not previously considered, like endoscopic resection of heterotopic ossification around the vertebral artery or endoscopic treatment of tethered cord. Readers will also be able to compare the technical options between monoportal and biportal endoscopic approaches, which are a frequent source of current debate and intrigue.

While these advances are both instructive and inspiring, questions remain for the reader to decide: Is this meaningful innovation? Will others follow the opportunities demonstrated herein? Are these surgeries only for a few surgeons? Are the benefits powerful enough to change the practices of others?

We hope you will find these cases illuminating and helpful in driving further advancement. The future is determined by the continued developments that boldly exceed our current expectations.

## Disclosures

Dr. Mahan reported personal fees from joimax and Arthrex; and grants from Boston Scientific, Renerva, and Nevro outside the submitted work. Dr. Snyder reported consulting fees from Globus Medical, Medtronic, DePuy, and Aesculap.
